# Two Draft Genome Sequences of a New Serovar of *Salmonella enterica*, Serovar Lubbock

**DOI:** 10.1128/genomeA.00215-15

**Published:** 2015-04-16

**Authors:** Marie Bugarel, Henk C. den Bakker, Kendra K. Nightingale, Dayna M. Brichta-Harhay, Thomas S. Edrington, Guy H. Loneragan

**Affiliations:** a International Center for Food Industry Excellence, Department of Animal and Food Sciences, Texas Tech University, Lubbock, Texas, USA; b U.S. Department of Agriculture, Agricultural Research Service, Roman L. Hruska U.S. Meat Animal Research Center, Clay Center, Nebraska, USA; c U.S. Department of Agriculture, Agricultural Research Service, Southern Plains Agricultural Research Center, Food and Feed Safety Research Unit, College Station, Texas, USA

## Abstract

*Salmonella enterica* is principally a foodborne pathogen that shows considerable serovar diversity. In this report, we present two draft genome sequences of *Salmonella enterica* subsp. *enterica* serovar Lubbock, a novel serovar.

## GENOME ANNOUNCEMENT

*Salmonella enterica* is a foodborne pathogen, showing considerable antigenic diversity with more than 2,500 serovars characterized to date (1, 2). This bacterium inhabits and colonizes a large variety of hosts and environments. Recent studies (3, 4) have highlighted the presence of *Salmonella* in bovine peripheral lymph nodes, which may be incorporated with meat to produce ground beef and therefore become a source of food contamination. We isolated atypical strains initially identified as serovar Montevideo. However, molecular interrogation of targeted genetic clades (5) revealed an atypical profile for Salmonella enterica serovar Montevideo. Further serological characterization of these isolates provided the antigenic formula of *S*. I. 6,7:g,m,s:e,n,z_15,_ representing a novel serovar that has been designated Lubbock. The strains sequenced in this announcement are 10TTU468 and 11TTU1590; both were isolated from subiliac lymph nodes from cattle at a commercial abattoir. Initial subtyping using multilocus sequence typing (6) or CRISPR locus content characterization showed subtypes previously associated with Salmonella enterica serovar Mbandaka.

Paired-end 151-bp reads were generated using an Illumina MiSeq platform. Reads were *de novo* assembled using the a5-pipeline version 20141120 (7), resulting in 38 and 36 contigs for 10TTU468 and 11TTU1590, respectively. The total assembly size was 4.95 Mbp for both strains, with *N*_50_ values of 263 kbp and 264 kbp and a median read depth of the assemblies of 85× and 74× for 10TTU468 and 11TTU1590, respectively. The draft genomes were annotated using the NCBI Prokaryotic Genome Automated Annotation Pipeline (8). Prophages were identified using PHAST (9). High-quality single nucleotide polymorphisms (hqSNPs) were called using software and parameters described previously by Den Bakker et al. (10), using the concatenated genome sequence of 10TTU468 as a reference, with rRNA and prophage regions excluded.

A total of 4,712 (10TTU468) and 4,709 (11TTU1590) protein-coding sequences were annotated in each genome. No plasmid-associated sequences were found, and both genome sequences contain seven intact prophages and five incomplete prophage regions. A kSNP-based (11) phylogenetic comparison using a representative variety of *S. enterica* serovars (12) and additional serovar Mbandaka isolates showed that both strains are closely related to *S. enterica* serovar Mbandaka 2009k-0807 (GenBank accession no. AMRS00000000.1) and 2012K-0273 (GenBank accession no. ARYT00000000.1). Further hqSNP-based comparison of the two isolates with 128 *S*. Mbandaka isolates publicly available from the NCBI Sequence Read Archive (SRA) (February 2015) showed that 10TTU468 and 11TTU1590 differ by 187 shared hqSNPs from the most closely related *S. enterica* serovar Mbandaka strain (NY_IDR1200021873-04, SRA accession no. SRX426108). Fifty-two hqSNPs mapped to a 4.5-kbp region containing the *fliC* gene. The high SNP density suggests homologous recombination within this region, and a BLASTn (13) search of the *fliC* sequences of Lubbock strains shows that this gene has a 100% identity to *fliC* in *S. enterica* serovar Montevideo strain 507440-20.

### Nucleotide sequence accession numbers. 

These whole-genome shotgun projects have been deposited in DDBJ/EMBL/GenBank under the accession numbers JXYU00000000 (10TTU468) and JXYV00000000 (11TTU1590). The versions described in this paper are JXYU01000000 and JXYV01000000.

## References

[1] Grimont P, Weill F 2007 Antigenic formulae of the *Salmonella* serovars: WHO Collaborating Centre for reference and research on *Salmonella*. Institut Pasteur, Paris, France.

[2] Guibourdenche M, Roggentin P, Mikoleit M, Fields PI, Bockemühl J, Grimont PA, Weill F-X 2010 Supplement 2003–2007 (no. 47) to the White-Kauffmann-Le Minor scheme. Res Microbiol 161:26–29. doi:10.1016/j.resmic.2009.10.002. 19840847

[3] Gragg SE, Loneragan GH, Brashears MM, Arthur TM, Bosilevac JM, Kalchayanand N, Wang R, Schmidt JW, Brooks JC, Shackelford SD, Wheeler TL, Brown TR, Edrington TS, Brichta-Harhay DM 2013 Cross-sectional study examining *Salmonella enterica* carriage in subiliac lymph nodes of cull and feedlot cattle at harvest. Foodborne Pathog Dis 10:368–374. doi:10.1089/fpd.2012.1275. 23566273 PMC3696922

[4] Gragg SE, Loneragan GH, Nightingale KK, Brichta-Harhay DM, Ruiz H, Elder JR, Garcia LG, Miller MF, Echeverry A, Ramírez Porras RG, Brashears MM 2013 Substantial within-animal diversity of *Salmonella* isolates from lymph nodes, feces, and hides of cattle at slaughter. Appl Environ Microbiol 79:4744–4750. doi:10.1128/AEM.01020-13. 23793628 PMC3719521

[5] Den Bakker HC, Moreno Switt AI, Govoni G, Cummings CA, Ranieri ML, Degoricija L, Hoelzer K, Rodriguez-Rivera LD, Brown S, Bolchacova E, Furtado MR, Wiedmann M 2011 Genome sequencing reveals diversification of virulence factor content and possible host adaptation in distinct subpopulations of *Salmonella enterica*. BMC Genomics 12:425. doi:10.1186/1471-2164-12-425. 21859443 PMC3176500

[6] Achtman M, Wain J, Weill F-X, Nair S, Zhou Z, Sangal V, Krauland MG, Hale JL, Harbottle H, Uesbeck A, Dougan G, Harrison LH, Brisse S 2012 Multilocus sequence typing as a replacement for serotyping in *Salmonella enterica*. PLoS Pathog 8:e1002776. doi:10.1371/journal.ppat.1002776. 22737074 PMC3380943

[7] Tritt A, Eisen JA, Facciotti MT, Darling AE 2012 An integrated pipeline for *de novo* assembly of microbial genomes. PLoS One 7:e42304. doi:10.1371/journal.pone.0042304. 23028432 PMC3441570

[8] Angiuoli SV, Gussman A, Klimke W, Cochrane G, Field D, Garrity G, Kodira CD, Kyrpides N, Madupu R, Markowitz V, Tatusova T, Thomson N, White O 2008 Toward an online repository of standard operating procedures (SOPs) for (meta)genomic annotation. Omics 12:137–141. doi:10.1089/omi.2008.0017. 18416670 PMC3196215

[9] Zhou Y, Liang Y, Lynch KH, Dennis JJ, Wishart DS 2011 PHAST: a fast phage search tool. Nucleic Acids Res 39:W347–W352. doi:10.1093/nar/gkr485. 21672955 PMC3125810

[10] Den Bakker HC, Allard MW, Bopp D, Brown EW, Fontana J, Iqbal Z, Kinney A, Limberger R, Musser KA, Shudt M, Strain E, Wiedmann M, Wolfgang WJ 2014 Rapid whole-genome sequencing for surveillance of Salmonella enterica serovar Enteritidis. Emerg Infect Dis 20:1306–1314. doi:10.3201/eid2008.131399. 25062035 PMC4111163

[11] Gardner SN, Hall BG 2013 When whole-genome alignments just won’t work: kSNP v2 software for alignment-free SNP discovery and phylogenetics of hundreds of microbial genomes. PLoS One 8:e81760. doi:10.1371/journal.pone.0081760. 24349125 PMC3857212

[12] Timme RE, Pettengill JB, Allard MW, Strain E, Barrangou R, Wehnes C, Van Kessel JS, Karns JS, Musser SM, Brown EW 2013 Phylogenetic diversity of the enteric pathogen *Salmonella enterica* subsp. *enterica* inferred from genome-wide reference-free SNP characters. Genome Biol Evol 5:2109–2123. doi:10.1093/gbe/evt159. 24158624 PMC3845640

[13] Altschul SF, Gish W, Miller W, Myers EW, Lipman DJ 1990 Basic local alignment search tool. J Mol Biol 215:403–410. doi:10.1016/S0022-2836(05)80360-2. 2231712

